# Chill coma recovery of *Ceratitis capitata* adults across the Northern Hemisphere

**DOI:** 10.1038/s41598-022-21340-y

**Published:** 2022-10-20

**Authors:** Cleopatra A. Moraiti, Eleni Verykouki, Nikos T. Papadopoulos

**Affiliations:** Laboratory of Entomology and Agricultural Zoology, Department of Agriculture Crop Production and Rural Environment, School of Agricultural Sciences, University of Thessaly, Fytokou St., 38446, Volos, Magnesia Greece

**Keywords:** Ecology, Ecophysiology, Entomology

## Abstract

The Mediterranean fruit fly, *Ceratitis capitata* (Diptera: Tephritidae), is an invasive pest, that is currently expanding its geographic distribution from the Mediterranean coasts to more temperate areas of Europe. Given that low temperature is a primary determinant of insect species’ range boundaries especially in the Northern Hemisphere with pronounced seasonality, we used chill coma recovery time for assessing latitudinal clines in basal chill tolerance of *C. capitata* adults. We selected six populations obtained from areas with broad climatic variability based on the main bioclimatic variables of temperature and precipitation, spanning a latitudinal range of about 19° from Middle East to Central Europe. Adults were exposed to 0 °C for 4 h, and time to regain the typical standing position of a fly at 25 °C were recorded. The post-stress survival after a period of 8 days was also recorded. Results revealed that adults from Israel and Austria were less chill tolerant than those from Greece, resulting in curvilinear trends with latitude. Analysis of macroclimatic conditions revealed combined effects of latitude (as a proxy of photoperiod) and macroclimatic conditions on chill coma recovery time. Nonetheless, there was not a deleterious effect on post-recovery survival, except for flies obtained from the northern most point (Vienna, Austria). Overall, it seems that evolutionary patterns of basal chill coma recovery time of *C. capitata* adults are driven mainly by local climatic variability.

## Introduction

In the Northern Hemisphere, the northward expansion of many terrestrial insects has been driven by climate warming and/or human-mediated transportation and trade^1–3^. Species with tropical or subtropical origin have been established in colder temperate regions, wherein they had to adapt to seasonal thermal variation and extreme weather events^4^. Hence, latitudinal clines in cold tolerance have been shaped in response to extreme winter minima overwinter survival^5,6^. Otherwise, evolutionary changes in the seasonal timing of life-history events, such as diapause termination, are expected to protect post-diapause adults from low temperatures that fall below critical thermal limits for activity during their active growth and reproduction periods^7^. In addition, chill-susceptible adults with increased chill tolerance can be protected from chilling injuries, which may have negative impact on fitness-related traits^8^. Performance at sub-lethal temperatures is therefore considered as a key limiting factor for population resistance and resilience at higher latitudes^9^. As a result, chill tolerance is a growing topic of research in an attempt to understand the species’ distribution limits with climatic variability, particularly for invasive insect species^2,10,11^.

One of the commonly measured chill tolerance trait is chill coma recovery time (CCRT), which refers to the time needed under benign conditions to recover neuromuscular function following a period of chill coma induced by temperatures that are commonly below the critical thermal limit for activity^12,13^. Prolonged exposure to low temperature causes a loss of ion balance and hemolymph hyperkalemia. High haemolymph [K^+^] can lead to chilling injuries, the effects of which cascade across tissues and may cause cell death (apoptosis) in the neuromuscular system; this process limits the ability of insects to recover, stand or fly, even if ion balance is restored. Recovery to warmer conditions involves both the rapid recovery of the temperature-induced depolarization and the energetically costly restoration of ion (and sometimes water) homeostasis, including upregulation of genes for repairing chilling injuries. Hence, the ability to quickly re-establishing homeostasis after cold stress directly affects the adaptation to low temperatures^14^. Sensitivity to such cold stress differ among population/insect species, and determines how fast individuals can conclude their foraging activities in order to feed, mate and/or escape from predators after a cold night and/or a frost event. Therefore, there are multiple potential fitness benefits from a fast recovery, suggesting that chill coma recovery can be a trait under selection^15^. To date, most efforts in understanding chill tolerance have focused on *Drosophila* spp. and/or *Drosophila* populations from tropical habitats that exhibit longer chill coma recovery time than those from temperate environments^5,6,10,16,17^, implying that chill coma recovery time can be a useful metric for disentangling inter- and intra-specific variation in chill tolerance^10^.

At the intraspecific level, the main selective pressures driving local adaptation in chill coma recovery time are inferred either from geographical proxies of environmental variation, such as latitude or altitude, or from a range of bioclimatic indicators of local climatic variability related mainly to temperature and precipitation. Linear latitudinal or altitudinal clines in chill coma recovery time have been observed for a few non-drosophilids, including the common woodlouse *Porcellio laevis*^18^, the winter ant *Prenolepis imparis*^19^ and the temperate-zone butterfly *Lycaena tityrus*^20^ with the high-latitude/altitude populations showing an increased resistance to cold, in line with the climatic variability hypothesis^21^. Colder environments are expected to harbor populations with higher chill tolerance in line with local climatic (thermal) variability and particularly the great variation in thermal minima across environmental gradients^22^. Chill coma recovery time was found to be correlated with daily minimum temperature in the Australian endemic species *Drosophila serrata*^5^, the minimum temperature at the coldest month for temperate and tropical *Drosophila melanogaster* populations from the coastal eastern Australia^6^, and both the annual mean temperature and annual mean minimum temperature for the common woodlouse, *P. laevis* from Chile^18^. In addition, Poikela et al. (2021) reported combined effects of latitude and bioclimatic variables on chill coma recovery time of *Drosophila flavomontana* adults by asserting that latitude is a proxy of photoperiod that serve as a reliable cue for seasonal temperature changes in the Northern Hemisphere. Chill coma recovery time of adults have evolved in response to latitudinal varying photoperiod but they are also associated with macroclimatic conditions of low-altitude coastal areas, wherein chill tolerance increases. Therefore, it seems that there is no ‘gold standard’ choice of environmental parameter for the relationship between insect chill tolerance and distribution limits^23^. A general assumption holds that latitude provides a better description of the geographical distribution while bioclimatic variables are key predictors of the thermal stress that limits performance and species’ persistence^24^.

The Mediterranean fruit fly (medfly), *Ceratitis capitata* (Wiedemann) (Diptera: Teprhitidae), is a highly damaging phytophagous pest with more than 300 host plant species, including cultivated trees of *Prunus* spp., *Citrus* spp. and *Pyrus* spp.^3,25^. It is an invasive pest, originated from eastern Sub-Saharan Africa, which has been dispersed in almost all tropical and sub-tropical regions of the world^3,26^. In the Northern Hemisphere, medfly has long been established in the Mediterranean Basin and Middle East but it is only recently that expanded its geographic distribution up to central Europe^27^. Since 2010, medfly adults have been captured in fruit-producing regions of Vienna, where seems to have been established ^27^. Even though medfly distribution is mainly attributed to anthropogenic activities based on transportation and/or trade, it is suggested that cold tolerance of *C. capitata* adults have jointly supported northward expansion of the species by facilitating population resistance to cold stress^28–33^. Thus far, evolutionary patterns of cold tolerance for *C. capitata* adults have been addressed only for some southern African populations in terms of critical thermal minima^32^.

Regarding chill coma recovery time of *C. capitata* adults, previous studies revealed that they are able to recover after a single short frost event in approximately 20 min (e.g. 0 °C for 1-4 h)^29–31,34^. At the intrapopulation level, flies with slower recovery time had reduced life expectancy, higher initial mortality rate, and worse climbing performance than their counterparts with faster recovery^30^. Nevertheless, only *C. capitata* flies that were reared for multiple generations under constant laboratory conditions have thus far been used for estimating chill coma recovery time. Eventhough lab-adaptation can result in rapid evolutionary changes in stress-related traits of insects^35^ (but see Popa-Báez et al., 2020^36^), any domestication effects on basal chill tolerance of *C. capitata* adults remain unexplored. Our preliminary data revealed slower recovery with artificial rearing under constant laboratory conditions for medfly adults from Greece than the wild flies (Figure S1 and Table S1 in Supplementary material). We recommend using wild flies than lab-adapted flies for assessing chill coma recovery time of *C. capitata* adults, and particularly for determining their geographical patterns of chill tolerance.

Here, we used chill coma recovery time as a chill tolerance metric for assessing evolutionary patterns of *C. capitata* adults from six populations spanning a latitudinal range of about 19°, from Middle East to Central Europe. Following a common-garden experimental approach, we used wild flies (up to F6 generation) from populations located at environmentally heterogenous habitats in order to be the most representative of the climatic variability faced at *C. capitata* habitats in the temperate zone across the Northern Hemisphere. Given the heterogeneity of climate based on Köppen-Geiger climate classification for the six fly collecting sites^37^, we initially quantified local climatic variability by performing a principal component analysis on the main bioclimatic variables of temperature and precipitation (in line with Poikela et al. (2021)). Then, we predicted that flies from the high-latitude, colder site in Central Europe will have lower chill coma recovery time than flies from the low-latitude, warmer site in Middle East, being in line with linear latitudinal clines reported previously for non-drosophilids species^18–20^. In an attempt to address the complex nature of the chill coma recovery time and distinguish whether latitudinal clines in chill tolerance of medfly adults have evolved in response to changes in photoperiod, macroclimatic conditions or their combination^38^, we assessed the effects of both local climatic conditions (based on bioclimatic variables) and latitude (as a proxy of photoperiod) on chill coma recovery time, accordingly to Poikela et al. (2021). Then, we assessed how chill coma recovery time, a non-lethal trait, accounts for geographic variation in post-recovery fitness, by estimating survival of both sexes for a period of 8 days under benign conditions. We predicted that populations with faster recovery will show higher survival than those with delayed recovery, in accordance with the previously reported intrapopulation variability in chill coma recovery time^30^. Considering that chill coma recovery time might be an important metric of performance under climatic variability, this study aims to provide insights regarding the recent northward expansion of medfly populations and a better understanding of population resistance after short frost events for making sound pest management decisions.

## Results

### Macroclimatic variability of the sites

The temperature-precipitation background of the six sites was characterized by principal component analysis (PCA) on 19 bioclimatic variables (see Supplementary Tables S2, S3). PCA revealed three principal components (PCs) with eigenvalues > 1. The first two PCs explained more than 86% of the total variation (see Supplementary Table S4) and were included in the candidate models for model selection.

**Figure 1:**
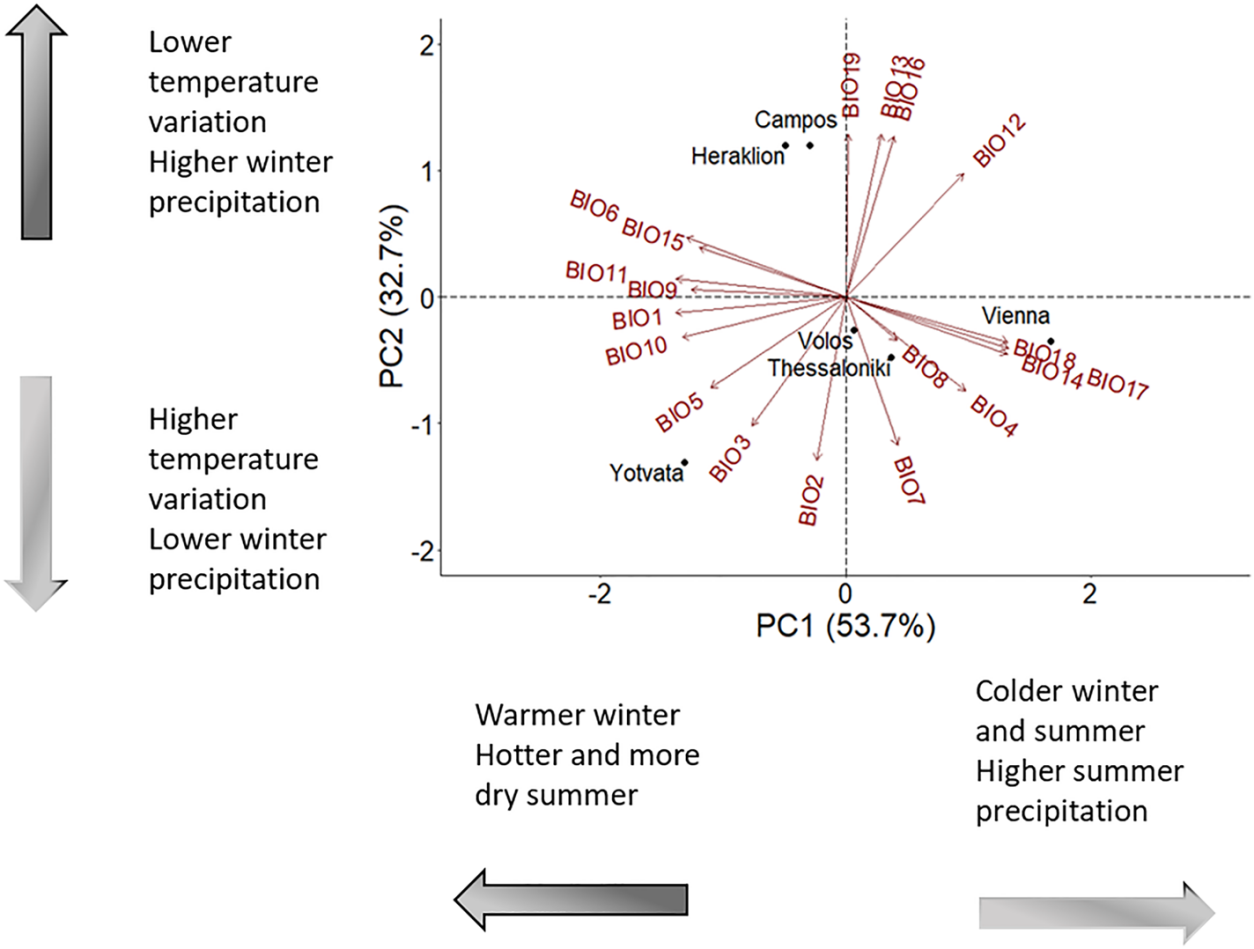
Climatic conditions in the six sampling sites. Principal component analysis (PCA) was performed on 19 variables describing environmental conditions in fly collecting sites.

**Figure 2:**
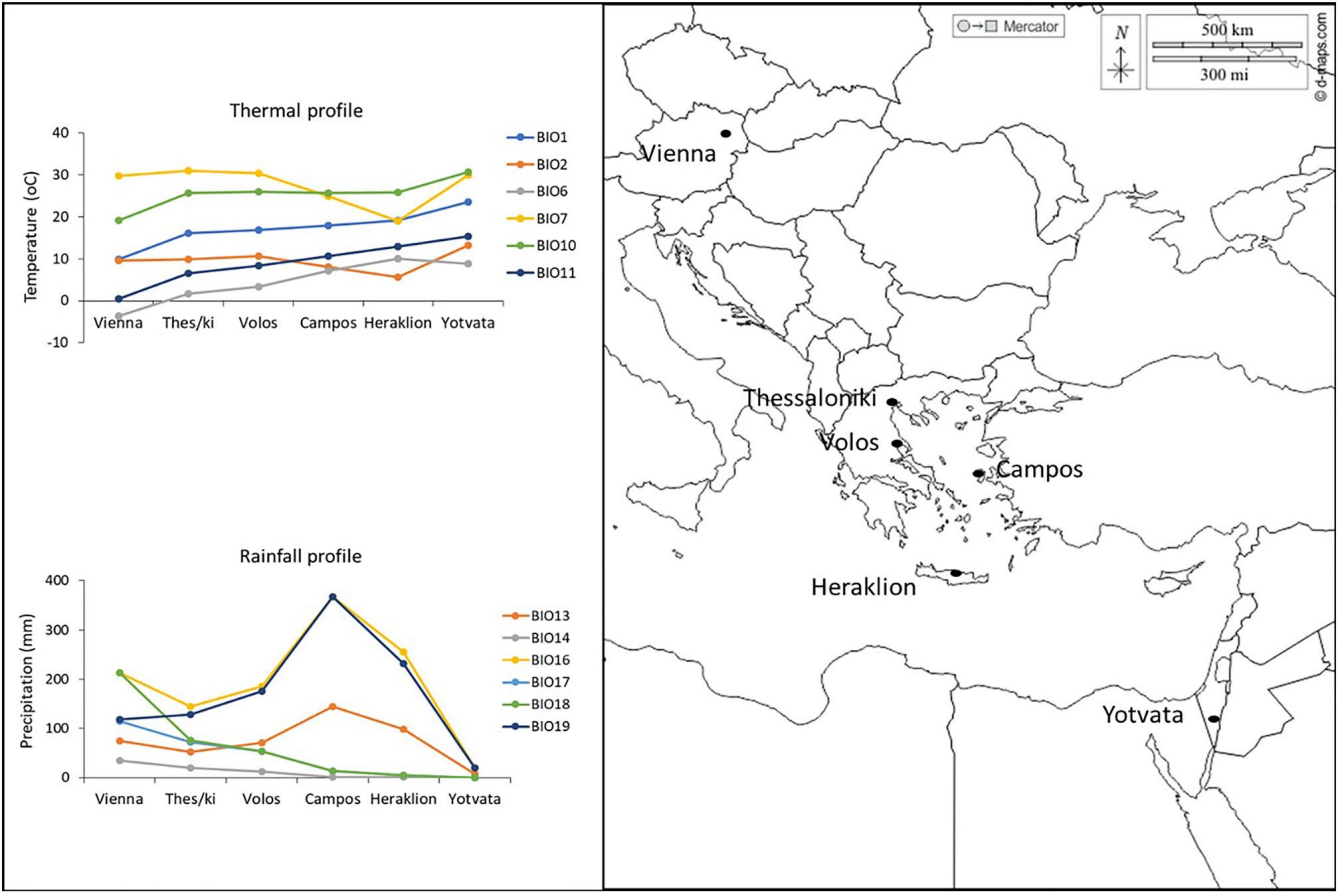
Map with the six collection sites for *Ceratitis capitata* populations. Climatic data of the six sites based on bioclimatic variables extracted from WordClim database (v2.1; current data 1970–2000; www.worldclim.org ) using latitudinal and longitudinal coordinates (0.5 min spatial resolution). Bioclimatic variables: Mean annual temperature (BIO1), mean diurnal range (BIO2), min temperature of coldest month (BIO6), temperature annual range (BIO7), mean temperature of the warmest quarter (BIO10), mean temperature of the coldest quarter (BIO11), precipitation of the wettest month (BIO13), precipitation of the driest month (BIO14), precipitation of the wettest quarter (BIO16), precipitation of the driest quarter (BIO17), precipitation of the warmest quarter (BIO18), precipitation of the coldest quarter (BIO19). The map template obtained from https://d-maps.com .

PC1 separated colder from warmer sites considering also the precipitation levels during summer (Fig. 1). Variables with the highest contribution on PC1 include annual mean temperature (BIO1), minimum temperature of coldest month (BIO6), mean temperature of warmest quarter (BIO10), mean temperature of coldest quarter (BIO11) and precipitation of driest month (BIO14), precipitation of driest quarter (BIO17) and precipitation of warmest quarter (BIO18) (see Supplementary Table S5). The high-latitude Vienna site is characterized by cold, relative wet winter and cold and wet summers (Fig. 2). On the other hand, temperature is high all year around in Yotvata (Israel) and summers are extremely dry. Average temperature during winter linearly increases with latitude, and the same trend is followed by minimum temperature of the coldest month, though minimum temperature is higher for Heraklion (Greece) than Yotvata (Fig. 2). Annual mean and summer temperatures did not differ among sites around the Mediterranean Basin (Fig. 2).

PC2 arranged the sites on the basis of daily and annual variability and winter precipitation. Variables with the highest contribution on PC2 include mean diurnal range (mean of monthly (max temp–min temp)) (BIO2), and temperature of annual range (BIO7) as well as precipitation of the wettest month (BIO13), precipitation of the wettest quarter (BIO16) and precipitation of the coldest quarter (BIO19) (see Supplementary Table S5). Thermal variability is low and precipitation high during winter in Herakleion and Campos sites (Chios, Greece) (Fig. 1). On the other hand, daily thermal variability is relative high in Yotvata, where precipitation during winter is extremely low (Fig. 2).

### Population-level variation in chill coma recovery time

**Figure 3:**
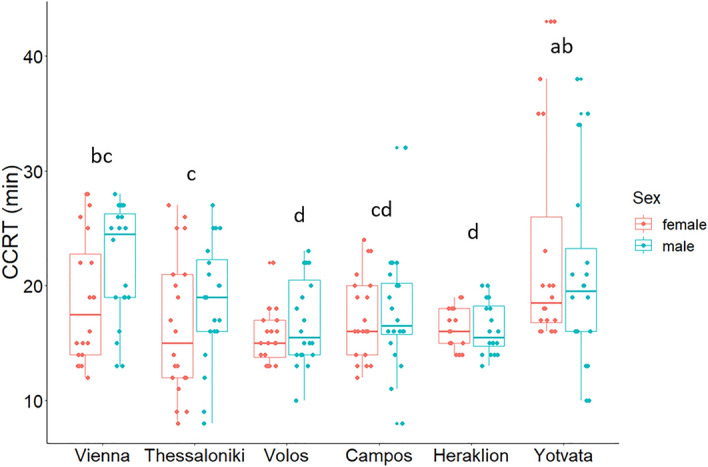
Chill coma recovery time (minutes) of males and females from the six *Ceratitis capitata* populations. Boxplots include the median, the 1st and 3rd quartile; whiskers indicate the highest/lowest value inside the interval defined by ± the 1.5-fold interquartile range from the 1st/3rd quartile. Populations labeled with the same lower case letter are not significantly different from each other (Benjamini-Hochberg (B-H) correction was used to adjust for multiple comparisons: *p* > 0.05). Sex was not significant (Cox Regression analysis, *p* = 0.142). N = 20 males and 20 females per population.

Average chill coma recovery time ranged from 22.1 ± 1.4 min (Yotvata) to 16.2 ± 0.5 min (Volos) for the six *C. capitata* populations (Fig. 3; see Supplementary Table S6). Cox Regression analysis revealed that population was a significant predictor of the chill coma recovery time (Wald’s *χ*^2^ = 44.093, df = 5, *p* < 0.001). Pairwise comparisons revealed that chill coma recovery time was longer for flies from Yotvata than those from the four Greek populations. Marginal differences in chill coma recovery time were recorded for flies from Campos and Heraklion (Fig. 3; see Supplementary Table S7). Flies from Yotvata and Vienna had similar chill coma recovery time. Within Greek populations, recovery was shorter for flies from Volos and Heraklion than those from Thessaloniki. Sex was not a significant predictor of the chill coma recovery time (Wald’s *χ*^*2*^ = 2.157, df = 1, *p* = 0.142).

#### Effect of latitude on chill coma recovery time

**Figure 4:**
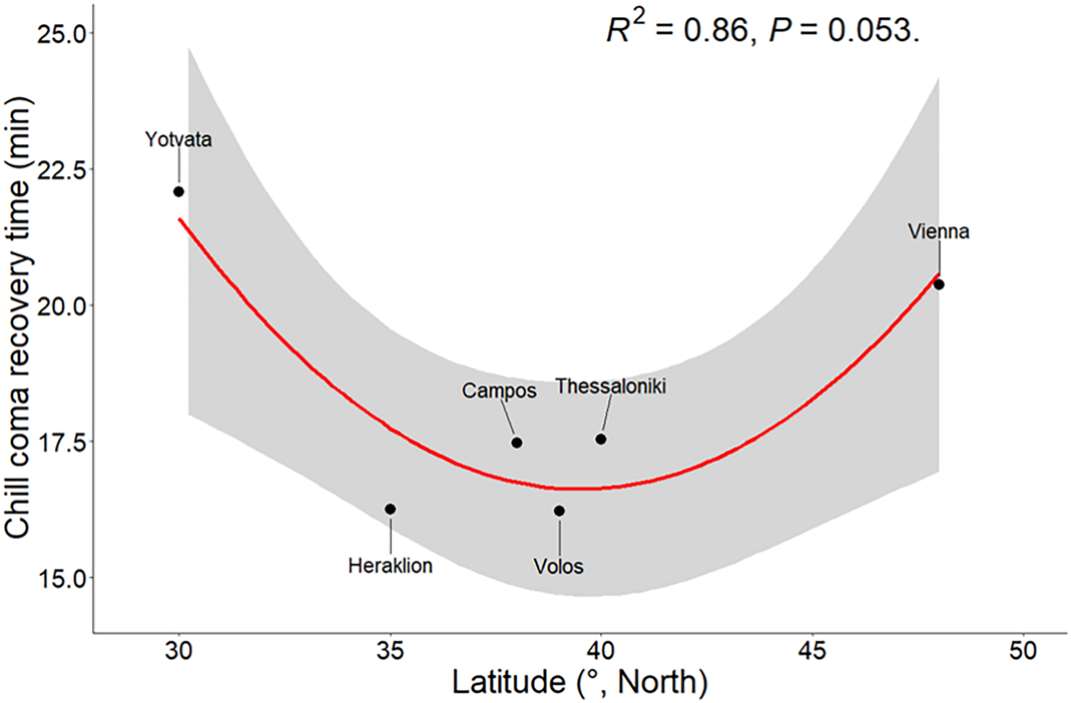
Latitudinal patterns in chill coma recovery time of *Ceratitis capitata* males and females from the six populations from the Northern Hemisphere. Points represent the mean CCRT for each population. There is a significant curvilinear relationship between chill tolerance and latitude (R^2^ = 0.86, *p* = 0.053, y = 102−4.35x + 0.055x^2^).

Regression analysis revealed a curvilinear relationship (quadratic, *R*^2^ = 0.86, *p* = 0.053) between chill coma recovery time and latitude, indicating increased chill tolerance between 30 and 40^o^N latitude, which represent the Greek populations, or otherwise the Mediterranean Basin (Fig. 4). In contrast, chill coma recovery time increases at both extremes of geographic distribution in the temperate climatic zone in the Northern Hemisphere.

#### Effects of bioclimatic variables on chill coma recovery time

**Figure 5:**
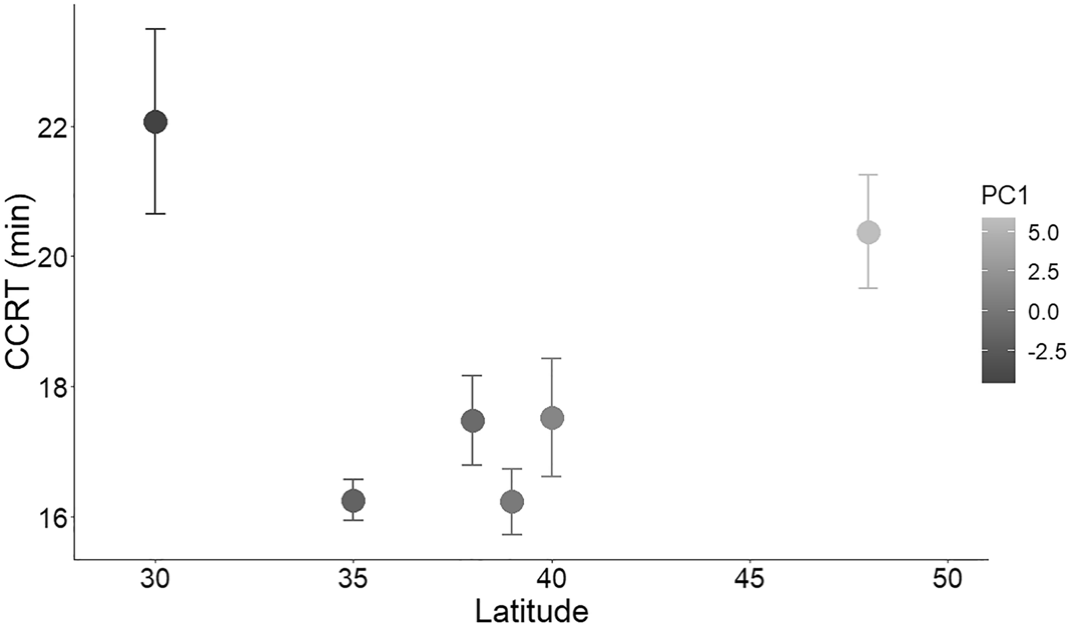
Relationship between latitude (as a proxy of photoperiod) and chill coma recovery time (CCRT) of *Ceratitis capitata* adults from six populations. The effects of latitudinally varying temperatures and summer precipitation (PC1) on *C. capitata* populations are illustrated in grey scale (lighter colors represents the colder populations with wet summers and the darker ones the warmer populations with dry summer). Error bars represent standard error of the mean (Mean ± SE).

The simplest model, which enabled us to distinguish between latitude (as proxy of photoperiod) and climatic variables, included latitude, PC1 and their interaction as explanatory factors (see Supplementary Table S8 for model comparisons). The best-fit model, carrying 30.7% of the cumulative model weight, revealed that macroclimatic conditions differ between the cold and warm sites (PC1) as well as the interaction of latitudinal varying photoperiod with the above macroclimatic conditions (latitude*PC1) are significant predictors of chill coma recovery time of *C. capitata* adults (Table 1, Fig. 5).

**Table 1:** Linear model results of the best model on the effects of latitude and/or climatic factors (PC1) on chill coma recovery time.

Model	Model parameters	B (SE)	Wald’s chi-squared test (df)	*p*-value
Latitude*PC1	Intercept	7.71 (21.7)	0.35 (1)	0.723
	Latitude	0.23 (0.56)	0.41 (1)	0.681
	PC1	−4.55 (1.46)	−3.11 (1)	**0.002**
	Latitude*PC1	0.10 (0.02)	5.36 (1)	**< 0.001**

Model selection was based on Bayesian Information Criterion (BIC) results (shown in Table S8). Significant *P*-values are shown in bold. *df*   degrees of freedom.

#### Recovery curves

**Figure 6:**
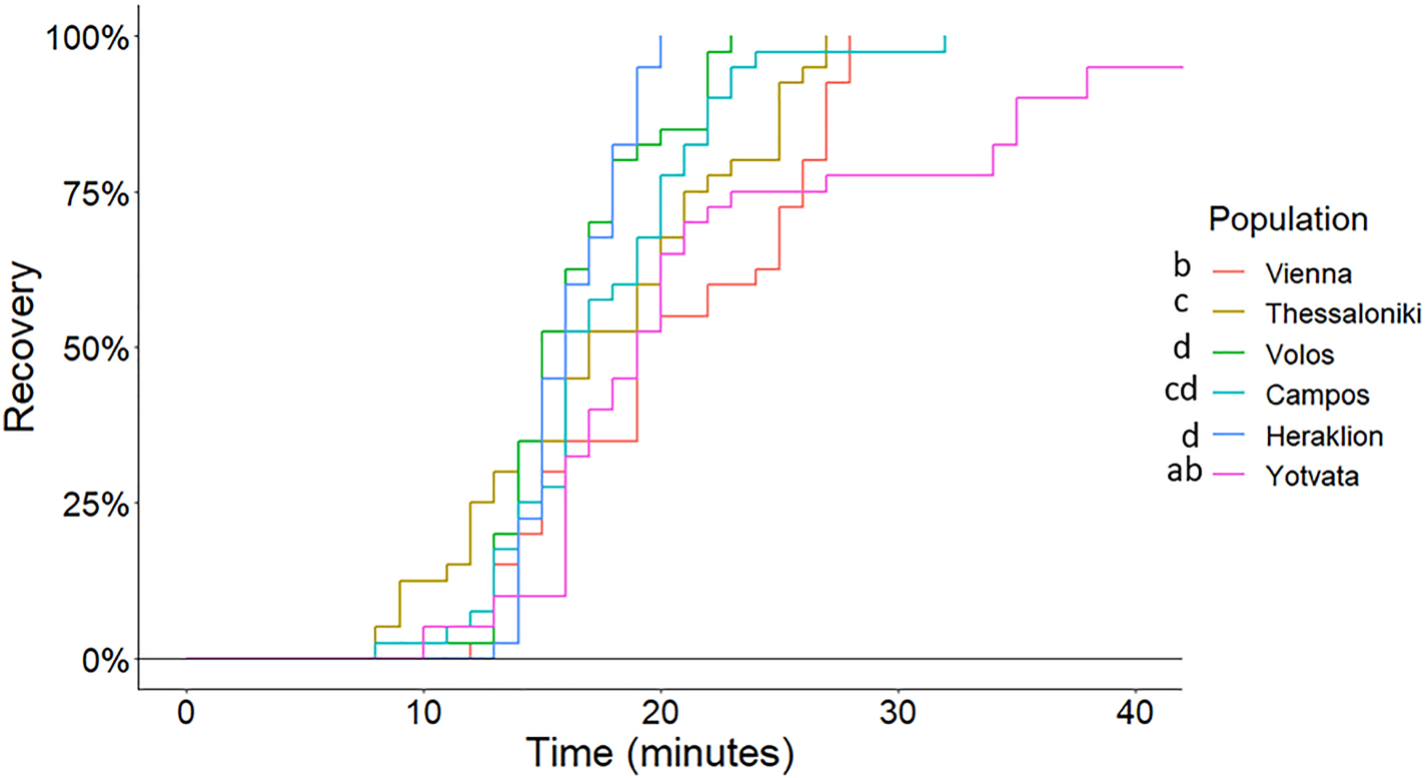
Kaplan–Meier recovery curves of adults from six *Ceratitis capitata* populations. Adults were exposed at 0 °C for 4 h and transferred for recovery at 25 °C. Populations labeled with the same lower case letter are not significantly different from each other (log rank test: *p* > 0.05). N = 40 adults per population.

Recovery rates were higher for flies from Greek populations than flies from Vienna and Yotvata (log-rank test, *p* < 0.001) (Fig. 6; see Supplementary Table S9). Fifty percent of flies from Greece had recovered within 15 min while recovery rates were progressively increased for flies from Yotvata (Fig. 6; see Supplementary Table S9). Recovery of flies from Heraklion and Volos were highly synchronized.

### Post-recovery survival

**Figure 7:**
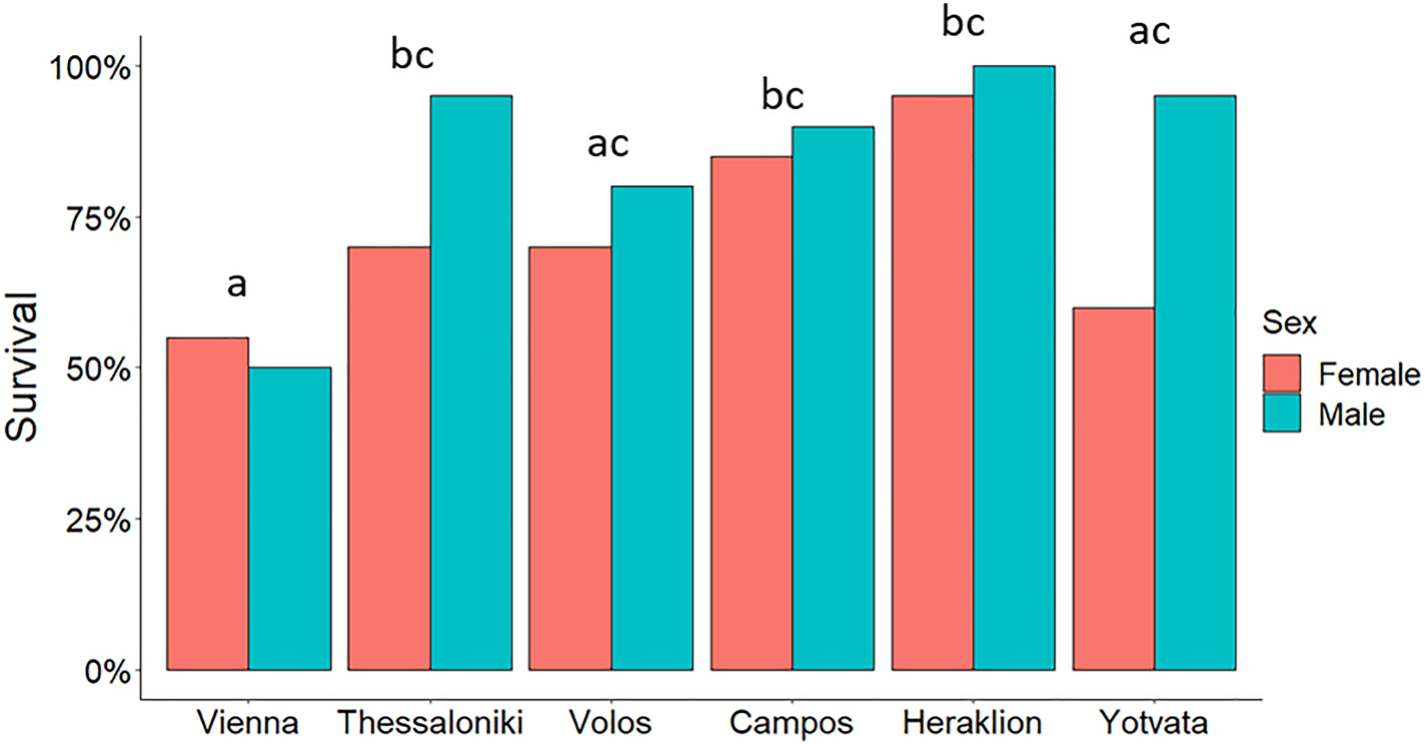
Post-recovery survival (%) of *Ceratitis capitata* adults from six populations. Adults were exposed to 0 °C for 4 h and survival of recovered flies were recorded after 8 days at 25 °C (with adult diet and water). Populations labeled with the same lower case letter are not significantly different from each other (Benjamini–Hochberg (B–H) correction was used to adjust for multiple comparisons: *p* > 0.05). Survival of females were lower than males (Logistic regression, *p* = 0.011). N = 20 males and 20 females per population.

Survivorship of the recovered flies after remaining 8 days at benign conditions ranged from 52.5% (Vienna) to 97.5% (Heraklion) among the six populations (Fig. 7). Logistic regression revealed that both population (*p* < 0.001) and sex (*p* = 0.011) were significant predictors of the post-recovery survival (see Supplementary Table S10).

Pairwise comparisons revealed that post-recovery survival of flies from the northernmost population of Vienna was lower than those of flies from all Greek populations, except from Volos. Post-recovery survival was marginally lower for flies from Vienna than that from Yotvata, which was marginally lower than those from Heraklion. Survivorship of flies from Heraklion was marginally higher than those from Volos (Fig. 7; see Supplementary Table S11). Survivorship of males was higher than that of females (*p* = 0.011).

## Discussion

This study examined the evolutionary responses in basal chill tolerance of *C. capitata* adults from six populations in the temperate zone across the Northern Hemisphere using a common-garden experimental approach. Despite geographic variation in chill coma recovery time, no linear latitudinal trends were found. Instead, a curvilinear relationship between chill coma recovery time and latitude was shaped, with a faster recovery for flies residing in the Mediterranean Basin. In an attempt to link the above latitudinal clines to macroclimatic conditions by using 19 bioclimatic variables, we found combined effects of regional climatic variability with latitude (as a proxy of photoperiod) on chill tolerance, underlying the complex nature of chill coma recovery time. Post stress survival was high for the recovered adults from all populations but Vienna. Survivorship of females was higher than those of males. It is seems, therefore, that chill coma recovery time of *C. capitata* adults is mainly driven by the local climatic variability of their habitas across the Northern Hemisphere.

Several studies have reported linear latitudinal clines in chill coma recovery time, particularly for drosophilids^5,6^. *Drosophila* species overwinter as adults and faster recovery from a cold stress provides fitness benefits during the cold season by increasing overwinter survival^18^. On the other hand, latitudinal trends of chill coma recovery time were absent across Australian populations of *Bactrocera tryoni*^36^ and *Bombus vosnesenskii* workers from Western United States^39^. In this study, *C. capitata* adults that reside in the Mediterranean Basin (35–40°) exhibited faster recovery than those from southern and northernmost populations, indicating a curvilinear relationship between chill coma recovery time and latitude across the Northern Hemisphere. One possible explanation for the low chill tolerance of Vienna adults is that populations near high-latitude range edges, especially if they are recent expansions, are likely to have phenotypes far from their local optimum because of higher genetic load^40^. Consistent with this expectation, populations from low-latitude range edges are also likely to perform more poorly than central populations across test sites, supporting the slower recovery of Yotvata flies as well. Moreover, recovery curves of adults from Yotvata and Vienna populations significantly differ from those from the Greek populations, validating a less steep, slower recovery with higher variability among individuals within populations. Even though the above patterns could be indicative of chilling injuries that results in lower fitness^30^, the increase in trait variance also increases the opportunity for selection under climatic stressful conditions^41^.

Curvilinear trends in chill coma recovery time were also demonstrated for the invasive widespread ant species *Myrmica rubra*, inhabiting sub-Arctic regions in the Northern Hemisphere, resulting from a connection with climates experienced by ancestral populations^11^. Given that gene flow is common among Greek populations^42^, ideally we could have controlled for population relatedness for excluding any phylogeographical connections, but genetic data were not currently available for all of the tested populations. On the other hand, the measurements of chill coma recovery time of Australian *D. melanogaster* populations revealed local adaptation to climatic selection along a latitudinal cline despite strong gene flow^43^. Thus, gene flow can either hinder or promote adaptation at range edges depending on the balance between the costs of migration and genetic drift^40,44^. Overall, considering the complicated interplay of selection, gene flow, and drift that affect evolutionary potential at range edges^40^, genetic studies are needed for elucidating the net effects of evolutionary forces on *C. capitata* populations that are resided in the Northern Hemisphere.

Bioclimatic indicators of each collection site can be a reliable indicator of climatic variability to address geographical variation in thermal selection^18^, albeit exceptions exist^23,39^. Latitude, which is not a real environmental variable, can serve as a proxy of photoperiod that is a more reliable cue for seasonal temperature changes than environmental temperature itself^23^. Presuming that chill coma recovery time is ecologically relevant to climatic variability^11,15^, an attempt was made to link chill coma recovery time of *C. capitata* adults with the macroclimatic conditions of the six sites. Model revealed that chill coma recovery time was significantly associated with PC1 and an interaction between latitude (as proxy of photoperiod) and PC1. On the one hand, the combinations of extreme temperature and precipitation (PC1) based on the annual mean temperature, the mean temperature of winter and summer seasons and the precipitation of the warmest season, which shape the climatic profile of the two climatic edge populations (namely the populations located at cold-climate Vienna and warm-climate Yotvata area), were associated with low chill tolerance. Temperature extremes, such as the minimum temperature of the coldest month that serves as proxy for the winter cold thresholds in each site, were also associated with chill tolerance in *C. capitata* flies, in line with drosophilids^6^. On the other hand, latitude found to be associated with the above bioclimatic variables (PC1) for estimating chill tolerance of *C. capitata* adults as well. This is probably due to site-specific differences in the seasonal availability of host fruits and the occurrence of frost events as well as differences in the overwintering capacity of *C. capitata* adults that regulate the duration of their flight period.

In Europe, relatively most frost events (when the daily minimum temperature drop below 0 °C) are expected in spring, particularly for populations around 40° latitude^45^. The day of the last spring frost as well as the phenological events have been advanced^46,47^, increasing the risk of exposure of the most vulnerable stage of insect life cycle and tree phenology to subsequent spring frosts. For example, the recent frost events of April 2016 and April 2017 caused crop losses in apple production in Austria^48^, which is the main fruit host of *C. capitata* in this area. In addition, late spring frost events are more severe to coastal areas compared with continental areas^47^. Moreover, *C. capitata* adults are more vulnerable to frost events (LLT50: 0 °C for 8 h)^33^ than immature stages (100% mortality after 7–9 days at 0 °C)^49^, resulting in geographical variation in overwintering capacity of medfly adults among the six site. Specifically, *C. capitata* overwinters as larvae (particularly 1st and 2nd instars) within fruits in Thessaloniki^50,51^ and Campos (Chios)^52^, while it overwinters in all stages in Crete due to mild winters^53^. Similarly, adults are captured all year round along the coastal plain and the Jordan Valley in central region of Israel^54^. In the area of Campos (Chios), adult flight period expand from June to January with peak captures from August to November^52^. In Volos, adult captures increases from May to November, but some adults may be captured until January (Papadopoulos & colleagues, unpublished data). In Thessaloniki, no adults are detected from December to the end of June, with increasing capture rates in autumn^25^. By contrast, the flight period is narrowed in Vienna, with most adult captures throughout August and September^27^. As a result, cold winters with low minimum temperatures are associated with adult absence from the coldest sites during the winter, which prevent them from being exposed to frost events during the coldest season when photoperiod is short (e.g. Vienna and Thessaloniki)^50^. In contrast, frost events are more often in autumn and winter for flies from latitudes around 30° (Yotvata) than in spring, as it is the case for flies in the Mediterranean Basin^45^. As a result, flies from the warmest area are on wings during seasons with short photoperiod when it is more likely to be exposed to frost events than during warmer seasons with long photoperiod. It is therefore, suggested to further assess the photoperiodic cues jointly to thermal cues for minimizing the chance of missing ecologically relevant patterns of basal chill tolerance in *C. capitata* flies.

The impact of sub-lethal stress on insect individuals may be of greater ecological importance than the ability to survive temperature extreme *per se*^55^. Nevertheless, latent chilling injury, which refers to cold-induced damage days after the stress^56^, has been rarely investigated and then often with contradictory results^57,58^. A recent meta-analysis revealed that survival is significantly decreased after extreme weather events, as opposed to reproduction and abundance^9^. Accordingly, post stress survival can be a useful proxy of population resistance after frost events for *C. capitata* flies. In this study, flies from all populations but Vienna demonstrated compensatory mechanisms during cold stress in order to reduce deleterious effects on survival. Even though both sexes need to adjust their physiology in order to survive a frost event^8^, the sex-related differences in post stress survival of *C. capitata* flies indicate that females are likely to shift a part of the investments into reproduction during the post-stress period, incurring survival costs. Nevertheless, all females that managed to recover were mated and reproductively mature and therefore, remaining alive for a period of 8 days after being exposed to chilling stress, gives them the opportunity to resume reproduction activities, and potentially increases population resistance. To this end, further studies are needed for determing the ability of the recovered females to reproduce effectively, by measuring their fecundity and fertility. Despite this limitation, this study contributes towards improving our understanding of how frost events during the adult life can affect long-term fitness of *C. capitata* flies and whether carry-over effects of frost events differ among *C. capitata* populations. The above knowledge could be useful both for predicting its distribution limits across the Northern Hemishere and for making sound pest management decisions in each area after a frost event. To this end, field validation of the results is a prerequisite for sustainable pest management decisions since laboratory may not provide transferable outcomes for pest management of *C. capitata*^59^.

Overall, this study is the first to address geographical patterns of chill coma recovery time of the adults of the widespread invasive pest *C. capitata*, revealing no linear latitudinal clines in the basal chill tolerance for populations residing in the temperate zone across the Northern Hemisphere. In the future, the use of more sampling sites, either within the same climate zone or from another climate zone met through the currently distribution range of *C. capitata* in the Northern Hemiphere, is highly recommended for excluding the possibility of alternative results under different sampling schemes. Moreover, a single frost event seems not to limit fitness of *C. capitata* flies in the Northern Hemisphere, but population resistence for flies from Vienna came under question. However, it is worth noting that chill coma recovery time is a plastic trait for *C. capitata* flies^29^, and it is therefore likely that flies from the relatively less chill tolerant populations to compensate their low basal chill tolerance with high cold acclimation capacity, as it is the case for other insects^60^. In this sense, the geographic patterns of developmental plasticity and adult acclimation on chill coma recovery time of *C. capitata* adults need to be addressed. It is also a need for further studies on seasonal variation in chill tolerance for multiple (≥ 3 years), at least for sites where *C. capitata* adults are on-winds all year around, as previous study reveals that chill coma recovery time of a natural population of *D. melanogaster* respond adaptively to seasonal shifts in temperature that are characteristic of temperate regions^61^. Last but not least, plasticity patterns of chill tolerance can be ideally combined with studies on their mechanistic base, for making sound predictions of the impact of climatic variability on population persistence and distribution^8^.

## Methods

### Populations

We used six populations that were originated across the temperate zone of the Northern Hemisphere. Populations were obtained from three countries: Austria (Vienna), Greece (Thessaloniki: northern Greece; Volos: central Greece; Campos: Chios Island; Heraklion: Crete Island) and Israel (Yotvata: Arabah). Population sampling sites spanning from ~ 29° to 48°N latitude with up to 157 m altitude in order to avoid altitudinal clines in chill tolerance^19,20^ (Fig. 2; see Supplementary Table S12). Accordingly to Köppen-Geiger climate classification^37^, the climate of Vienna is classified as temperate oceanic (Cfb), with the average temperature of the warmest month being below 22 °C, the coldest averaging above 0 °C, and at least four months with average above 10 °C. There are no strict seasonal patterns of precipitation. All Greek populations but Thessaloniki have a typical hot summer Mediterranean climate (Csa), with at least one month’s average temperature above 22 °C, four months above 10 °C, and the coldest above 0 °C. Winter is the wettest period while the driest month of summer receives less than 40 mm. The climate in Thessaloniki is classified as cold semi-arid (Bsk), and it is characterized by cold, relative wet winters and hot dry summers. Israel lies in a transition zone between the hot and arid southern part of West Asia and the relatively cooler and wetter northern Mediterranean region. Yotvata belongs to one of the three dryland zones in southern part that is characterized by an extremely hyper-arid climate (Bwh)^62^. Summers are hot and totally dry, following by mild winters with low average annual rainfall that greatly varies from year to year. Hence, Yotvata population represents a climatic edge population in the temperate zone of the Northern Hemisphere while Vienna could be characterized as a climatic edge population located at the highest latitude of the current distribution.

### Insect rearing

Pupae were retrieved from field infested fruits (peaches, oranges, apples and pomegranate) from the six sampling sites, from late summer to early winter based on the local availability of infested host fruits. Collected fruits were transferred to laboratory, placed in plastic containers on a layer of sterilized sand and remained under standard conditions (25 ± 1 °C, 60 ± 5% relative humidity and 14:10 L:D photoperiod) until pupae collection. The collected pupae from different sites were used to raise separate, site-specific populations/colonies. Wild adults (F_0_) (N = 500–2000 individuals were retrieved from the infested fruits under the above standard laboratory conditions at University of Thessaly (UTH), AT-AGES and Agricultural Research Organization (ARO) for Greek, Austrian and Israeli populations respectively. Flies were allocated at 4 cages in each generation, and after rearing for 1–2 generations in fruits under standard laboratory conditions, pupae from Austria (F_2_) and Israel (F_1_) were delivered by a courier agent to UTH. Upon emergence, adults kept in wooden (30 × 30 × 30 cm), wire-screened cages provided with water and a standard adult diet (yeast hydrolysate, sugar, and water at 1:4:5 ratio). All cages were held at similar low densities (approx. 150 individuals) and females were allowed to oviposit on 5-cm-diameter hollow, plastic hemispheres of red color (domes) that were artificially punctured with 40–50 evenly distributed holes on their surface. Each dome was fitted in a 5-cm-diameter hole made on the cover of a 5.5-cm-diameter plastic petri dish. Water was placed in the base of the petri dish in order to maintain humidity levels (beneath the dome) adequate enough for female oviposition. A plastic cup containing 0.5 ml of orange juice was placed in the base of the petri dish to stimulate oviposition.

All flies were reared for three to four generations in the UTH laboratory conditions before being used for the chill coma recovery assays. Specifically, we used flies reared up to F_6_ (F_3_, F_4,_ F_4_, F_4_, F_5_, and F_6_ for Heraklion, Thessaloniki, Volos, Campos, Yotvata and Vienna population respectively) for avoiding maternal, trans-generational or other epigenetic effects of field populations as well as laboratory adaptation issues that may raise under prolonged rearing conditions^63^. The above common garden approach will provide evidence that observed phenotypic differences are not environmentally induced and help to identify the role of local selective factors^64^.

### Chill Coma Recovery Time (CCRT) assays

Based on previous results found that an exposure of *C. capitata* lab-adapted adults at 0 °C for 4 h is sufficient to induce chill coma and cause variability in recovery time among individuals within a population^30,31^, we predicted that recovery from the same cold stress will provide a clear-cut discrimination among the six *C. capitata* populations. In addition, we used 10-day-old adults to control for any potential age-related differences in chill tolerance, which can markedly influence trait assessments^65^.

To determine chill coma recovery time (CCRT), we used 20 males and 20 females for each population. Upon emergence, adults from each population were placed into Plexiglas cages (20 × 20x20cm) with ad libitum access to adult diet food and water. On adult day 10, groups of 8–10 mixed-sex adults of the same population were transferred into empty 35-mL glass vials with a cotton wool stopper. Vials were immersed in an ice-water slurry at 0 °C for 4 h (in the dark) in a Styrofoam cooler box placed at room temperature (25 °C). The temperature within vials were checked by placing an analog thermometer into an empty glass vial immersed into the ice-water slurry. Following chill coma, flies were immediately placed individually in petri dish (5 cm in diameter) in a supine position (using a paintbrush), and their recovery was monitored for one hour under laboratory conditions (25 ± 1 °C, 60 ± 5% relative humidity). Petri dishes were sealed with a transparent plastic lid to prohibit escape of recovered flies. A fly was scored as recovered when it was able to right itself and stand on its legs in a normal posture or fly, without any interference or stimulation from the observer. The time period needed at 25 °C until reach the upright position was termed “chill coma recovery time”^13^. There were no dead flies observed during chill coma recovery time assays (0 °C for 4 h) or censored flies (flies that remained alive but did not reach an upright position for 1 h after being transferred at 25 °C for recovery from chill coma assay).

### Post recovery survival

For each population, flies that recovered from chill coma assays were transferred back into their Plexiglas cages (by gently pushing them from their individual petri dish into a Plexiglas cage with a paintbrush, in rarely cases when they did not fly directly into their cage). All flies had ad libitum access to adult diet and water and remained under standard laboratory conditions (25 ± 1 °C, 60 ± 5% relative humidity, 14L:10D) for the next 8 days. Dead males and females were recorded daily. Post-recovery survival of males and females were calculated as the percentage of the recovered males and females that remained alive 8 days after the chill coma assay.

### Statistics

Statistical analysis was conducted using R version 4.1.1 (R Development Core Team 2021, R Foundation for Statistical Computing, Vienna, Austria)^66^. To characterize macroclimatic conditions of the six collection sites, we extracted 19 bioclimatic variables (Bioclim1-19) related to temperature and precipitation from WorldClim database (Version 2.1, www.worldclim.org) using latitudinal and longitudinal coordinates for each site (0.5 min spatial resolutions; current data 1970–2000)^67^ (Table S3, Supplementary material). A principal component analysis (PCA) on 19 bioclimatic variables was performed to investigate environmental variation across fly populations. Model selection was performed using the “bicab” and “aicab” functions of package “AICcmodavg”^68^. Then, the Bayesian Information Criterion (BIC) model selection was used to distinguish among a set of possible linear regression models describing the relationship of latitude (as a proxy of photoperiod), PC1, PC2 on chill coma recovery time. BIC uses a stronger penalty for including additional variables to the model. The general linear model was used for the parameter estimation of the best-fit model with meaningful biological meaning. The relationship of chill coma recovery time with latitude was initially examined using a linear regression model but the model fit was inadequate (R^2^ = 0.009) so a second-degree polynomial model was adopted. A Cox proportional hazards regression (packages *survival*^69^, *survminer*^70^) was applied to model recovery times adjusting for population, sex, and their interactions. Kaplan–Meier curves and pairwise log-rank tests (package *emmeans*^71^) were used for the comparison of populations’ recovery rates. A logistic regression model was used to examine the effect of population and sex on post-recovery survival. Statistically non-significant interactions were removed by the model. Benjamini-Hochberg (B-H) correction was used to adjust for multiple comparisons in both Cox proportional hazards and logistic regression models. P-values less than 0.05 were considered statistically significant.

## Supplementary Information

Supplementary Information.

## Data Availability

All data will become available up-one request and will be uploaded in an open access folder of the FF-IPM project.
