# Supplementary material for: Chill coma recovery of Ceratitis capitata adults across the Northern Hemisphere
Source: Sci Rep. 2022 Oct 20;12:17555. doi: 10.1038/s41598-022-21340-y (PMC9585097; doi:10.1038/s41598-022-21340-y)
Supplement: Supplementary file 1 — Supplementary Information. [file 41598_2022_21340_MOESM1_ESM.docx]

**SUPPLEMENTARY INFORMATION**

**Supplementary Table S1**. Results of Cox Regression analysis for the effects of generation and sex on chill coma recovery time of *Ceratitis capitata* adults from Volos (Thessaly, Greece). Flies emerged from infested citrus that were collected from the same location during the years 2020 and 2021. Flies reared under standard laboratory conditions up to F1 (2021 collection), F4 (2021 collection) and F13 (2020 collection). Ten days-old flies were exposed at 0^o^C for 4h and transferred at 25^o^C until recovery. Significant differences are indicated with bold. N=25-40 flies for each generation. Male flies from F13 generation form the baseline.

|  | B (SE) | Wald’s chi-squared test (df) | p-value |
| --- | --- | --- | --- |
| Generation |  | 15.133 (2) | 0.001 |
| F1 | 0.315 (0.257) | 1.499 (1) | 0.221 |
| F4 | -0.614 (0.264) | 5.385 (1) | 0.020 |
| Sex | 0.276 (0.203) | 1.852 (1) | 0.174 |

**Supplementary Table S2**. List of 19 bioclimatic variables used in the PCA (WorldClim database v2.1, 0.5 min spatial resolutions; current data 1970-2000[^1^](#_ENREF_1);[www.worldclim.org](http://www.worldclim.org) ).

| Variable | Description | | | |
| --- | --- | --- | --- | --- |
| BIO1 | Annual Mean Temperature |  |  |  |
| BIO2 | Mean Diurnal Range (Mean of monthly (max temp - min temp)) | | | |
| BIO3 | Isothermality (bio2/bio7) (×100) | |  |  |
| BIO4 | Temperature Seasonality (standard deviation ×100) | | |  |
| BIO5 | Max Temperature of Warmest Month | |  |  |
| BIO6 | Min Temperature of Coldest Month | |  |  |
| BIO7 | Temperature Annual Range (bio5-bio6) | |  |  |
| BIO8 | Mean Temperature of Wettest Quarter | |  |  |
| BIO9 | Mean Temperature of Driest Quarter | |  |  |
| BIO10 | Mean Temperature of Warmest Quarter | |  |  |
| BIO11 | Mean Temperature of Coldest Quarter | |  |  |
| BIO12 | Annual Precipitation |  |  |  |
| BIO13 | Precipitation of Wettest Month | |  |  |
| BIO14 | Precipitation of Driest Month |  |  |  |
| BIO15 | Precipitation Seasonality (Coefficient of Variation) | | |  |
| BIO16 | Precipitation of Wettest Quarter | |  |  |
| BIO17 | Precipitation of Driest Quarter |  |  |  |
| BIO18 | Precipitation of Warmest Quarter | |  |  |
| BIO19 | Precipitation of Coldest Quarter | |  |  |

**Supplementary Table S3.** 19 bioclimatic variables for each site were extracted from WorldClim database v2.1 using latitudinal and longitudinal coordinates (0.5 min spatial resolutions; current data 1970-2000[^1^](#_ENREF_1); [www.worldclim.org](http://www.worldclim.org) )

| Population | Long. | Lat. | BIO1 | BIO2 | BIO3 | BIO4 | BIO5 | BIO6 | BIO7 | BIO8 | BIO9 | BIO  10 | BIO  11 | BIO  12 | BIO13 | BIO14 | BIO15 | BIO16 | BIO17 | BIO  18 | BIO19 |
| --- | --- | --- | --- | --- | --- | --- | --- | --- | --- | --- | --- | --- | --- | --- | --- | --- | --- | --- | --- | --- | --- |
| Vienna | 16.3 | 48 | 9.9 | 9.6 | 0.32 | 72.5 | 26.1 | -3.7 | 30 | 19 | 1.8 | 19.2 | 0.5 | 623 | 75 | 35 | 25 | 213 | 115 | 213 | 118 |
| Thes/ki | 22.9 | 40 | 16.1 | 9.8 | 0.31 | 74.3 | 32.6 | 1.6 | 31 | 8.4 | 25 | 25.7 | 6.6 | 440 | 53 | 20 | 27 | 144 | 72 | 76 | 128 |
| Volos | 22.9 | 39 | 16.9 | 10.6 | 0.34 | 69.3 | 33.7 | 3.3 | 30 | 10 | 26 | 26 | 8.3 | 491 | 71 | 12 | 43 | 186 | 54 | 54 | 176 |
| Campos | 26.1 | 38 | 17.9 | 8 | 0.32 | 59.6 | 32 | 7.1 | 25 | 11 | 26 | 25.7 | 10.7 | 624 | 145 | 1 | 89 | 367 | 14 | 14 | 367 |
| Heraklion | 25.1 | 35 | 19.1 | 5.6 | 0.29 | 50.4 | 29 | 10 | 19 | 13 | 26 | 25.8 | 12.9 | 514 | 98 | 1 | 79 | 255 | 5 | 5 | 232 |
| Yotvata | 35.06 | 29 | 23.5 | 13.2 | 0.44 | 59.3 | 38.7 | 8.8 | 30 | 15 | 31 | 30.7 | 15.4 | 35 | 8 | 0 | 95 | 20 | 0 | 0 | 20 |

**Supplementary Table S4.** Principal Components (PCs) with their Eigenvalues, variance and cumulative variance.

| PC | Eigenvalue | Variance (%) | Cumulative Variance (%) |
| --- | --- | --- | --- |
| PC1 | 3.194 | 53.7 | 53.7 |
| PC2 | 2.494 | 32.74 | 86.44 |
| PC3 | 1.342 | 9.48 | 95.92 |
| PC4 | 0.866 | 3.94 | 99.87 |
| PC5 | 0.156 | 0.13 | 100 |

**Supplementary Table S5.** Contributions (loadings) of the altitude and 19 bioclimatic variables on the Principal Components (PC).

|  | PC1 | PC2 | PC3 | PC4 | PC5 |
| --- | --- | --- | --- | --- | --- |
| BIO1 | 9.7 | 0.1 | 0 | 0.2 | 1.6 |
| BIO2 | 0.3 | 13.7 | 0.5 | 14.1 | 8.3 |
| BIO3 | 2.9 | 8.5 | 2.6 | 16.3 | 4.2 |
| BIO4 | 4.8 | 4.5 | 12.3 | 0.8 | 10.3 |
| BIO5 | 6.1 | 4.2 | 4.5 | 4.4 | 0.3 |
| BIO6 | 8.5 | 1.8 | 0.6 | 1 | 0.7 |
| BIO7 | 0.9 | 11.3 | 7.9 | 9 | 0.2 |
| BIO8 | 0.9 | 1 | 45.7 | 3.7 | 1.3 |
| BIO9 | 8 | 0 | 9.3 | 1.8 | 2 |
| BIO10 | 8.9 | 0.9 | 1.9 | 0.1 | 1.6 |
| BIO11 | 9.6 | 0.2 | 0.3 | 0.3 | 0.4 |
| BIO12 | 4.7 | 7.8 | 1.3 | 1.1 | 28.8 |
| BIO13 | 0.4 | 13.7 | 0.8 | 12.1 | 0.4 |
| BIO14 | 8.7 | 1.7 | 0 | 0.8 | 9.5 |
| BIO15 | 7.2 | 1.2 | 5.5 | 11.2 | 27.8 |
| BIO16 | 0.8 | 13.4 | 0.6 | 10.7 | 0.3 |
| BIO17 | 8.9 | 1.4 | 0.5 | 0.2 | 0.3 |
| BIO18 | 8.8 | 1 | 2 | 0.4 | 1.6 |
| BIO19 | 0 | 13.6 | 3.6 | 11.8 | 0.4 |

**Supplementary Table S6.** Chill coma recovery time (minutes) for *Ceratitis capitata* adults from six populations from the temperate climatic zone in the Northern Hemisphere. Flies (10d-old) were exposed at 0^o^C for 4h and then remained at 25^o^C until recovery. Data for males and females are pooled.

| Population | CCRT (min) | |
| --- | --- | --- |
|  | mean ± SE | (min, max) |
| Vienna | 20.1 ± 0.9 | (12,28) |
| Thessaloniki | 17.5 ± 0.9 | (8,27) |
| Volos | 16.2 ± 0.5 | (10,23) |
| Campos | 17.5 ± 0.7 | (8,32) |
| Heraklion | 16.3 ± 0.3 | (13,20) |
| Yotvata | 22.1 ± 1.4 | (10,43) |

**Supplementary Table S7.** Pairwise comparisons in chill coma recovery time for *Ceratitis capitata* adults from six populations originating from the temperate climatic zone in the Northern Hemisphere. Adults were exposed at 0^o^C for 4h and transferred for recovery at 25^o^C. Benjamini-Hochberg (B-H) correction was used to adjust for multiple comparisons. Significant differences are indicated with bold.

| Contrast | | ratio(SE) | z | p |
| --- | --- | --- | --- | --- |
| Vienna | Thessaloniki | 0.656 (0.148) | -1.866 | 0.085 |
| Vienna | Volos | 0.380 (0.089) | -4.13 | **<0.001** |
| Vienna | Campos | 0.561 (0.129) | -2.517 | **0.022** |
| Vienna | Heraklion | 0.348 (0.084) | -4.392 | **<0.001** |
| Vienna | Yotvata | 1.388 (0.339) | 1.343 | 0.207 |
| Thessaloniki | Volos | 0.579 (0.132) | -2.391 | **0.028** |
| Thessaloniki | Campos | 0.855 (0.192) | -0.695 | 0.522 |
| Thessaloniki | Heraklion | 0.530 (0.124) | -2.712 | **0.014** |
| Thessaloniki | Yotvata | 2.116 (0.524) | 3.026 | **0.006** |
| Volos | Campos | 1.478 (0.333) | 1.732 | 0.104 |
| Volos | Heraklion | 0.916 (0.207) | -0.388 | 0.698 |
| Volos | Yotvata | 3.657 (0.932) | 5.087 | **<0.001** |
| Campos | Heraklion | 0.620 (0.142) | -2.09 | 0.055 |
| Campos | Yotvata | 2.475 (0.614) | 3.651 | **0.001** |
| Heraklion | Yotvata | 3.992 (1.033) | 5.352 | **<0.001** |

**Supplementary Table S8.** Model selection for chill coma recovery time of *Ceratitis capitata* adults based on Bayesian Information Criterion (BIC). The model selection included Latitude and PC2, PC1 as well as different interaction terms. Bayesian Information Criterion weight (BICWt), i.e. probability of being the best model, was chosen for further analysis.

| Model | Κ | AICc | AICcWt | BIC | BICwt | Log-Likelihood |
| --- | --- | --- | --- | --- | --- | --- |
| PC1*PC2 | 5 | 1502.30 | 1.22 x 10^-1^ | 1519.45 | 3.23 x 10^-1^ | -746.023 |
| latitude*PC1 | 5 | 1502.41 | 1.16 x 10^-1^ | 1519.56 | 3.07 x 10^-1^ | -746.076 |
| latitude*PC2+PC1 | 6 | 1499.68 | 4.52 x 10^-1^ | 1520.21 | 2.22 x 10^-1^ | -743.661 |
| latitude*PC1+latitude*PC2 | 7 | 1501.74 | 1.62 x 10^-1^ | 1525.62 | 1.48 x 10^-2^ | -743.627 |
| latitude*PC1+PC2 | 6 | 1502.02 | 1.40 x 10^-1^ | 1522.55 | 6.87 x 10^-2^ | -744.831 |
| latitude*PC2 | 5 | 1508.09 | 6.73 x 10^-3^ | 1525.24 | 1.78 x 10^-2^ | -748.920 |
| PC2 | 3 | 1513.24 | 5.15 x 10^-4^ | 1523.58 | 4.11 x 10^-2^ | -753.567 |
| PC1+PC2 | 4 | 1515.19 | 1.94 x 10^-4^ | 1528.95 | 2.80 x 10^-3^ | -753.511 |
| latitude+PC2 | 4 | 1515.22 | 1.91 x 10^-4^ | 1528.97 | 2.77 x 10^-3^ | -753.523 |
| latitude+PC1+PC2 | 5 | 1517.15 | 7.26 x 10^-5^ | 1534.30 | 1.93 x 10^-4^ | -753.449 |
| null | 2 | 1527.80 | 3.54 x 10^-7^ | 1534.71 | 1.57 x 10^-4^ | -761.875 |
| latitude+PC1 | 4 | 1527.91 | 3.36 x 10^-7^ | 1541.66 | 5.00 x 10^-6^ | -759.869 |
| latitude | 3 | 1529.52 | 1.50 x 10^-7^ | 1539.87 | 1.20 x 10^-5^ | -761.712 |
| PC1 | 3 | 1529.748 | 1.34 x 10^-7^ | 1540.09 | 1.10 x 10^-5^ | -761.823 |

K: The number of parameters in the model.

AICc: The information score of the model.

AICcWt: Probability of being the most parsimonious model among the candidate model set.

BIC: The Bayesian information criterion for each model

BICWt: Probability of being the most parsimonious model among the candidate model set.

Log-Likelihood: The log-likelihood of each model.

BICWt of the best-fit and biologically meaningful model is shown in grey.

**Supplementary Table S9.** Pairwise comparisons (Kaplan Meier, log rank test) in chill coma recovery time of *Ceratitis capitata* adults from six populations originating from the temperate climatic zone in the Northern Hemisphere. Adults were exposed at 0^o^C for 4h and transferred for recovery at 25^o^C. Benjamini-Hochberg (B-H) correction was used to adjust for multiple comparisons. Significant differences are indicated with bold. N=20 males and 20 females per population.

|  | Thessaloniki | Volos | Campos | Heraklion | Yotvata |
| --- | --- | --- | --- | --- | --- |
| Vienna | **0.025** | **<0.001** | **0.015** | **<0.001** | 0.249 |
| Thessaloniki | - | **0.044** | 0.419 | **0.014** | **0.031** |
| Volos |  | - | 0.133 | 0.605 | **<0.001** |
| Campos |  |  | - | 0.018 | **0.014** |
| Heraklion |  |  |  | - | **<0.001** |

**Supplementary Table S10.** Results of the analysis of deviance table on the logistic regression of survivorship against population and sex. *Ceratitis capitata* adults from six populations were exposed to 0^o^C for 4h and then recovered flies remained at 25^o^C for 8 days. Survivorship were estimated as percentage of the recovered flies that remained alive after 8 days. N=20 males and 20 females per population.

|  | df | Deviance | Resid. df | Resid. deviance | Pr(>Chi) |
| --- | --- | --- | --- | --- | --- |
| Population | 5 | 28.696 | 234 | 219.58 | <0.001 |
| Sex | 1 | 6.413 | 233 | 213.17 | 0.011 |

**Supplementary Table S11.** Pairwise comparisons of survivorship against population and sex. *Ceratitis capitata* adults from six populations were exposed to 0^o^C for 4h and then recovered flies remained at 25^o^C for 8 days. Survivorship were estimated as percentage of the recovered flies that remained alive after 8 days. Benjamini-Hochberg (B-H) correction was used to adjust for multiple comparisons. Significant differences are indicated with bold. N=20 males and 20 females per population.

| Contrast | | OR(SE) | z | p |
| --- | --- | --- | --- | --- |
| Vienna | Thessaloniki | 0.222 (0.118) | -2.824 | **0.024** |
| Vienna | Volos | 0.353 (0.174) | -2.107 | 0.075 |
| Vienna | Campos | 0.148 (0.086) | -3.274 | **0.008** |
| Vienna | Heraklion | 0.026 (0.028) | -3.416 | **0.008** |
| Vienna | Yotvata | 0.306 (0.154) | -2.347 | 0.057 |
| Thessaloniki | Volos | 1.593 (0.895) | 0.829 | 0.509 |
| Thessaloniki | Campos | 0.667 (0.428) | -0.631 | 0.609 |
| Thessaloniki | Heraklion | 0.118 (0.129) | -1.946 | 0.097 |
| Thessaloniki | Yotvata | 1.381 (0.788) | 0.566 | 0.612 |
| Volos | Campos | 0.419 (0.255) | -1.428 | 0.230 |
| Volos | Heraklion | 0.074 (0.079) | -2.409 | 0.057 |
| Volos | Yotvata | 0.867 (0.464) | -0.267 | 0.789 |
| Campos | Heraklion | 0.176 (0.198) | -1.544 | 0.204 |
| Campos | Yotvata | 2.069 (1.277) | 1.174 | 0.326 |
| Heraklion | Yotvata | 11.729 (12.733) | 2.268 | 0.058 |

**Supplementary Table S12**. Geographic characteristics of the six *Ceratitis capitata* populations. The climate of each fly collection site is classified accordingly to Köppen-Geiger climate classification[^2^](#_ENREF_2).

| Population | Longitude | Latitude | Altitude (m, a.s.l.) | Local climatic zone |
| --- | --- | --- | --- | --- |
| Vienna | 16.3 | 48 | 151 | Cfb (temperate oceanic) |
| Thessaloniki | 22.9 | 40 | 157 | Bsk (cold semi-arid) |
| Volos | 22.9 | 39 | 5 | Csa (hot summer Mediterranean) |
| Campos | 26.1 | 38 | sea level | Csa (hot summer Mediterranean) |
| Heraklion | 25.1 | 35 | 33 | Csa (hot summer Mediterranean) |
| Yotvata | 35.06 | 29 | 70 | Bwh (desert hot arid) |

**
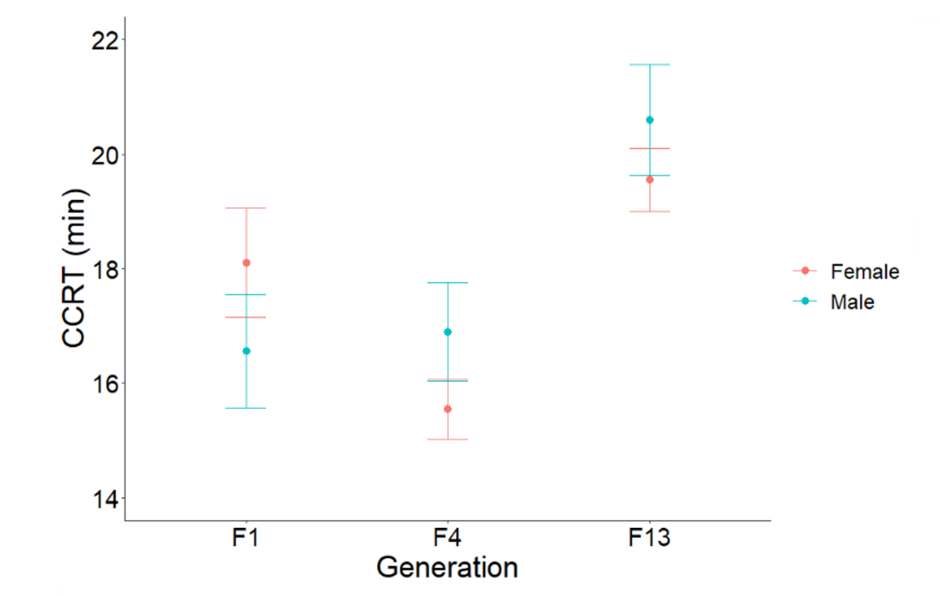
**

**Supplementary Figure S1.** Chill coma recovery time (mean ± SE) of *Ceratitis capitata* males and females from Volos (Thessaly, Greece). Flies emerged from infested citrus that were collected from the same location during the years 2020 and 2021. Flies reared under standard laboratory conditions up to F1 (2021 collection), F4 (2021 collection) and F13 (2020 collection). Ten days-old flies were exposed at 0^o^C for 4h and transferred at 25^o^C until recovery. Ν=25-40 flies for each generation.

**References**

1 Fick, S. E. & Hijmans, R. J. WorldClim 2: new 1‐km spatial resolution climate surfaces for global land areas. *International journal of climatology* **37**, 4302-4315 (2017).

2 Beck, H. E. *et al.* Present and future Köppen-Geiger climate classification maps at 1-km resolution. *Scientific data* **5**, 1-12 (2018).
